# Influence of the Arrangement of Mechanical Fasteners on the Static Strength and Fatigue Life of Hybrid Joints

**DOI:** 10.3390/ma13235308

**Published:** 2020-11-24

**Authors:** Marek Rośkowicz, Jan Godzimirski, Andrzej Komorek, Jarosław Gąsior, Michał Jasztal

**Affiliations:** 1 Department of Mechatronics, Armament and Aerospace, Military University of Technology, 00-908 Warsaw, Poland; jan.godzimirski@wat.edu.pl (J.G.); michal.jasztal@wat.edu.pl (M.J.); 2 Department of Aviation, Polish Air Force University, 08-530 Dęblin, Poland; a.komorek@law.mil.pl; 3 Military Centre for Standardization, Quality and Codification, 00-909 Warsaw, Poland; j.gasior@ron.mil.pl

**Keywords:** hybrid joint, static strength, fatigue life, fastener

## Abstract

This paper presents the results of experimental research and numerical calculations regarding the static strength and fatigue life of hybrid joints. In the experiments, specimens built as single-lap adhesive–mechanical joints (hybrid joints) were tested. In a two-stage process of the failure of the hybrid joints, the adhesive joint was damaged first. Therefore, it was assumed that the assembly of fasteners closer to the edge of the overlap (beyond the ranges recommended for mechanical joints) limits the negative impact of the peeling phenomenon on the strength and performance properties of hybrid joints. The specimens used in the experiments were prepared from composite elements (i.e., carbon fiber-reinforced polymer (CFRP)), as well as from the aluminum alloy 2024T4. Because the detection of fatigue damage in composite materials is a complex problem, computed tomography was used to evaluate the degradation of the composite material. Experimental and numerical comparative analyses of the static strength and fatigue life of hybrid joints with adhesive and mechanical joints confirmed the assumptions made.

## 1. Introduction

The pursuit of aviation transport constructors, especially of aircraft, to lower the weight has led to the replacement of a very large number of designed metal components with composite elements. Composite structures are joined in three different ways—by using mechanical joints [1,2], by means of an adhesive [3], or by a combination of these two methods [4,5].

Hybrid joints are a combination of adhesive joints and mechanical fasteners (e.g., rivets and bolts). Those joints build a structural node and are often simpler and cheaper to realize than other traditional joining methods [6]. In the case of the simultaneous use of both rivets/bolts and adhesive joints to form a hybrid joint, it is possible to emphasize their benefits and to mitigate the disadvantages of both types of joints [7,8] when used separately. Due to the increasing use of polymer composite materials in aircraft or in cars, the performance of this type of joint, as well as their mechanical properties, including the load capacity and fatigue life, are taken into consideration [9,10]. The load capacity and fatigue life of hybrid joints depend on several construction, technological, and material parameters, including the type of joint and the adhesives and rivets used [11,12,13]. For example, the viscoelasticity of adhesives has a positive effect on the fatigue life of joints loaded with a one-sided cycle, because during such loading, the maximum stresses at the joint edge are reduced [14]. In addition, rivets in this type of joint limit an emergence of sudden unexpected damage [9,15], which is crucial in aviation constructions. Very often, a combination of these types of joint is exploited to improve the characteristics and performance of the construction node, also ensuring the sealing of the joint or protecting a construction from the adverse effects of weather conditions.

In the adhesive layer of a hybrid joint, similar to in adhesive joints, there are problems associated with non-uniform stress distribution in the bonded layer. Since in both bonded and hybrid joints, there is often a complex load condition, apart from a non-uniform shear stress field [16], in adhesive joints there is also a non-uniform distribution of normal stresses perpendicular to the surface of bonded elements, resulting in an unfavorable peeling phenomenon. The highest values of normal stresses, similar to shear stresses, occur on lap joint edges [17,18]. Therefore, fasteners are introduced on lap joint edges to significantly reduce the normal stresses and to increase the strength and performance parameters, including their carrying capacity and fatigue life.

In mechanical joints, it is possible to use different numbers and distribution patterns of fasteners (e.g., rivets and bolts); however, in every case, they weaken the structure of the connected elements [19]. This problem is more relevant in the case of layered composites, in which delamination initiators are constituted by the holes for fasteners and material loading through surface pressures. In the process of connecting composites, apart from mechanical joints, the additional use of an adhesive further reduces the number of rivets. Moreover, hybrid joints generally demonstrate higher static strength [20,21,22,23,24] and fatigue strength [9,20] rather than separately using adhesive or mechanical joints. Experimental studies show that hybrid joints demonstrate the best mechanical properties when significant elements of loading are evenly carried either by rivets or an adhesive joint [22,25,26,27]. The results of previous research [21,22,24] indicate, however, that only a limited number of examined joints achieve this condition, in which the load is evenly shared by the two types of joints, mainly due to the fact that an adhesive undergoes earlier failure and the joint then acts as a mechanical one. Furthermore, in study [28], it was found that the loads are evenly carried over only a small area. The research [29] also shows that placing the mechanical connector in the middle of the joint does not increase the strength of the joint—the joint is failed first, and then the connector. This problem was analyzed in [30] by changing the spacing of mechanical connectors in the hybrid connection of a composite material with an aluminum alloy sheet. It is worth noting that the load capacity of hybrid joints can also be increased by using washers between the fastener head and the joined material, thus increasing the area of the fastener’s positive influence on the stress distribution in an adhesive joint [20].

A number of studies have assumed using an adhesive joint as a connection supporting a rivet so as to reduce the number of rivets. They have also tested the phenomena that occur in adhesive joints, which additionally contain rivets, and it has been shown that depending upon the configuration, they either have no effect or may improve the mechanical properties of hybrid joints [29,31].

In this article, the authors attempted to assess the effect of using fasteners in adhesive joints on the load capacity and fatigue life of the created hybrid joints. In order to reduce the negative phenomena of stress concentration on lap joint edges, the location of mechanical fasteners was modified. The reference point to locate fasteners at a distance that is recommended for mechanical joints is two-hole diameters of the rivets/bolts (2d) from the edge of the lap. In the modified hybrid joints, the fasteners were fixed at a distance equal to one diameter (1d). It was expected that such a solution would primarily reduce the value of the stresses that cause the peeling phenomenon on lap joint ends, thus delaying the formation of cracks and delamination, which otherwise lead to the failure of the joint [32]. Reducing stress values at lap joint edges should result in increasing the load capacity and fatigue life of hybrid joints. During the study, elements made of carbon fiber-reinforced polymer (CFRP), as well as of the aluminum alloy type 2024T4, were used.

In order to qualitatively assess the influence of the displacement of fasteners closer to the overlap edge, hybrid joint research using the finite element method (Ansys Workbench version 19, ANSYS Inc., Canonsburg, PA, USA) was carried out.

## 2. Methodology

### 2.1. Numerical Calculations

The aim of the numerical calculations was to test the hypothesis that the distribution of mechanical connectors in hybrid joints affects the normal stresses in the adhesive layer, causing it to peel, and thus impacting the strength of joints and their durability. In adhesively bonded lap joints, these stresses are greater than shear stresses and are the cause of failure of the adhesive layer. In hybrid connections, the failure usually takes place in two stages—the adhesive layer fractures first, then the mechanical fasteners. Reducing the value of the normal stresses that cause peeling, through the appropriate location of mechanical fasteners, should delay the failure of the adhesive layer and thus increase the static strength and fatigue life of a hybrid joint. The calculations were performed for metal elements, because, taking into account the qualitative nature of the numerical calculations, their repetition for composite materials was considered redundant. A simple numerical model was used in the calculations because the purpose of the calculations was only to perform a qualitative check of the effect of the spacing of mechanical connectors on the value of the normal stresses that cause peeling of the adhesive layer. The model took into account the pressures generated by the heads of mechanical fasteners. The calculations were carried out using the Static Structural module of ANSYS v.19.2 software.

For computational purposes, a model of a hybrid single lap joint specimen was prepared with two mechanical fasteners mounted at the ends of the overlap. Because in the next stage of the experimental research, it was planned to use joints with a 50 mm overlap, calculations were also made for this length of overlap. In terms of the properties of the materials used, the following conditions were adopted:

(a) Bilinear properties of aviation aluminum alloy 2024 for adherends;
(b) Linear properties of Epidian 57/Z1 adhesive;
(c) Linear properties of structural steel for mechanical fasteners.

The values of these parameters adopted for the calculations are presented in Table 1.

**Figure 1:**
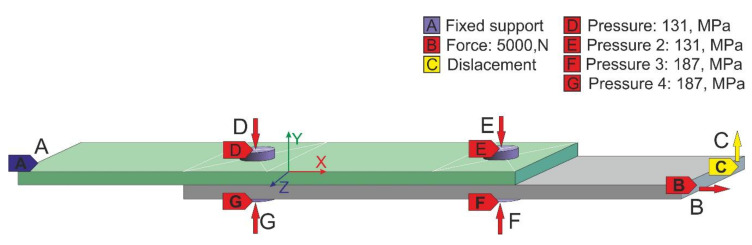
Boundary conditions assumed for the tested hybrid joint specimen. **A**—fixed support, **B**—force, **C**—displacement, **D**–**G**—pressures.

The joined specimens were cuboid-shaped and measured 75 × 25 × 2 mm. An adhesive joint thickness of 0.1 mm was used for the calculations. Two calculation cases were analyzed, changing the position of the mechanical fasteners in relation to the edges of the connection overlap. In the first case, the location of the fasteners was equal to two diameters of the 2d fasteners (which meant that the axis of the hole for the fastener was 6 mm away from the edge of the lap) and one diameter (hole axis was 3 mm away from the edge of the lap). In the boundary conditions of the model, the load range also included the surface pressure (Figure 1D–G) that occurred as a result of the installation of the mechanical fasteners. In addition, the displacement of one end of the model in a normal direction (Figure 1C) to the adhesive layer by 2 mm was also included to reflect the assembly in the testing machine holder for experimental purposes, considering both bending moments and joint initial load. The fasteners were tightened (2.2 Nm) considering the value of torque; this reflects a force of 3000 N, which was implemented (using adequate pressure) on the head and sleeve modeling the mechanical fastener nut. In addition, the free end of the model was loaded with a force of 5000 N (Figure 1B) directed perpendicularly to the cross-section of the joint element, while the other end of the specimen was constrained to avoid any displacement or rotation (Tx, Ty, Tz, Rx, Ry, and Rz = 0). The boundary conditions mentioned above are presented in Figure 1.

The hybrid joint was modeled as an object comprised of more than one element; therefore, a definition of contact was necessary. The defined contact elements among the specimen, adhesive, and fasteners are shown in Table 2.

**Figure 2:**
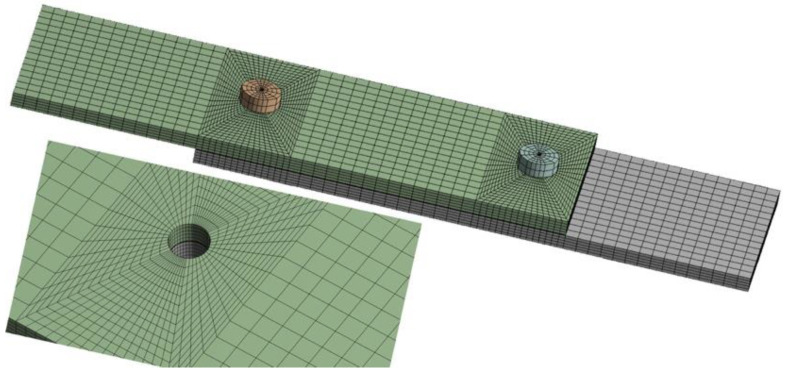
Hexagonal mesh created for the numerical research.

The value of the friction coefficient *f* was adopted, defining friction in global terms, giving its average value [33]. The adhesive layer was modeled with one layer of elements, while the specimens were divided into five layers. For modeling, a hexagonal mesh was used, which was refined in the vicinity of the fasteners’ holes (Figure 2). The model created for the numerical research consisted of 28,792 elements (140,946 nodes).

### 2.2. Experimental Research

In order to confirm the observations from the numerical research regarding the positive effect of arranging the mechanical fasteners closer to the edge of the overlap in hybrid joints, experimental studies were performed.

Specimens of CFRP and aluminum alloy, adhesive, mechanical, and hybrid (mechanical-adhesive) joints were used. The dimensions of the connected rectangular-shaped elements were 100 × 25 × 2 mm (length/width/thickness) for the aluminum alloy and 100 × 25 × 2.2 mm for the CFRP. The lap surface was 50 × 25 mm (adhesive).

**Figure 3:**
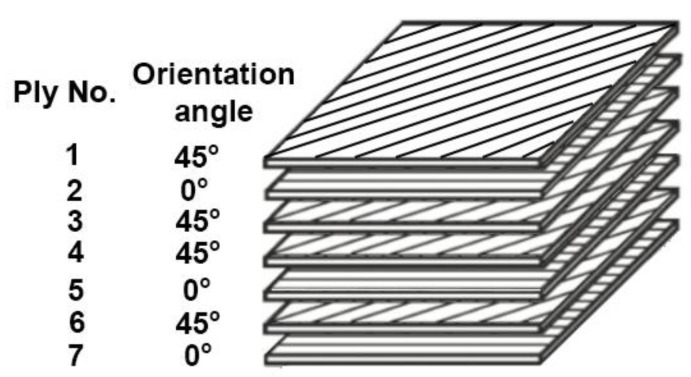
Scheme of the distributing layers in the composite material.

The CFRP elements were prepared by WaterJet cutting technology from panels made in autoclaves, on the basis of DF285 prepreg. The scheme of the subsequent layers in the composite is presented in Figure 3.

The CFRP was cured using the following thermal cycle: Holding rise of 2 °C/min until 120 °C, holding at a temperature of 120 °C for 90 min, cooling 2 °C/min to a temperature of 40 °C.

**Figure 4:**
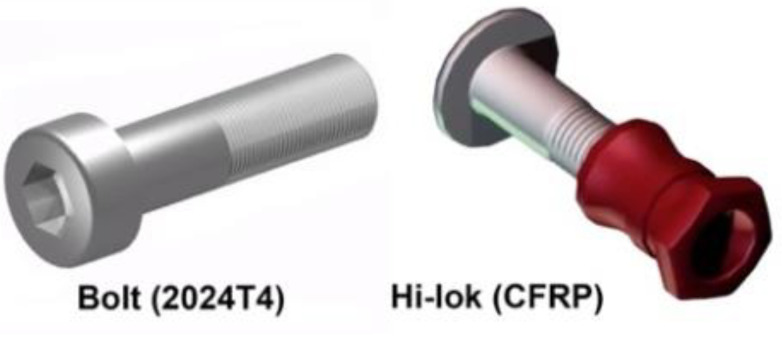
The fasteners used in the assembly of the metal and composite samples.

The adhesive and hybrid joints were bonded with Epidian 57/Z1 Sarzyna manufactured by Ciech S.A. The adhesive was cured in two stages, i.e., for 24 h at a temperature of 20 °C and for 8 h at a temperature of 80 °C. The mechanical and hybrid joints were prepared as follows: For the aluminum alloy samples, steel bolts were used (series 8.8, 3 mm in diameter, BN-73/1112-03) and for the composite specimens, Hi-lok series HL 1012 fasteners (Hi-Shear Technology Corporation, Torrance, CA, USA) were used (Figure 4). The holes prepared for the bolts were 3 mm in diameter, while for the Hi-loks, the diameter was 4.1 mm. The lengths of the grooved fasteners were selected in such a manner that the assembly holes did not have grooving cuts.

The bonded surfaces of the metal and composite elements were degreased with acetone. The surfaces of the aluminum alloy specimens, in order to increase the mechanical adhesion, were additionally sanded with sandpaper with a P80 grit size (abrasive grain size ranging from 180 to 212 μm). The surfaces of the CFRP elements were pre-roughened in order to break the peel ply. The mechanical fasteners (i.e., bolts and Hi-loks) were fixed halfway through the width of the specimens in two different configurations in terms of the distance of the fastener from the edge of the lap. The distance was expressed through the diameter of the holes for the fasteners—2d or 1d of the hole diameter. The bolts were tightened to a torque of 2.2 Nm. The specimens were subjected to static loads by a Zwick/Roell Z100 machine (ZwickRoell, Ulm, Germany) and to fatigue loads by an Instron 8802 machine (Instron, Norwood, MA, USA). The studies of the fatigue life, depending on the type of sample material (i.e., metal or composite), the joining methods (i.e., adhesive, mechanical, or hybrid) and the assembly configuration of the mechanical fastener (i.e., 2d or 1d) in the load range are reflected in Table 3.

In the first stage of the research, load capacity, fatigue life of the adhesive, and mechanical and hybrid joints of aluminum alloy specimens were compared. In the second stage, the load capacity and fatigue life of the joints prepared from the CFRP were also compared. Two variants of assembly using mechanical fasteners were studied (i.e., 1d and 2d) for the aluminum alloy as well as the CFRP specimens. This methodology was used purposefully to estimate the impact of these modifications on the load capacity and the fatigue life of the joints.

Tests of the load capacity of the joints were conducted for a series of five samples, whereas for fatigue life (due to longevity), tests were designated for series of three to four specimens. Because the detection of fatigue damage in composite materials is a complex problem, the method of computed tomography was used to evaluate the degradation of the composite material. The CT scanner manufactured by GE v/tome/x m was exploited in the studies.

**Figure 5:**
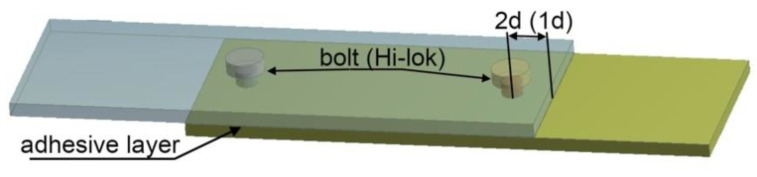
A hybrid joint specimen.

A hybrid joint specimen used in the research is presented in Figure 5.

## 3. Results and Analysis

### 3.1. Results and Discussion of Numerical Calculations

**Figure 6:**
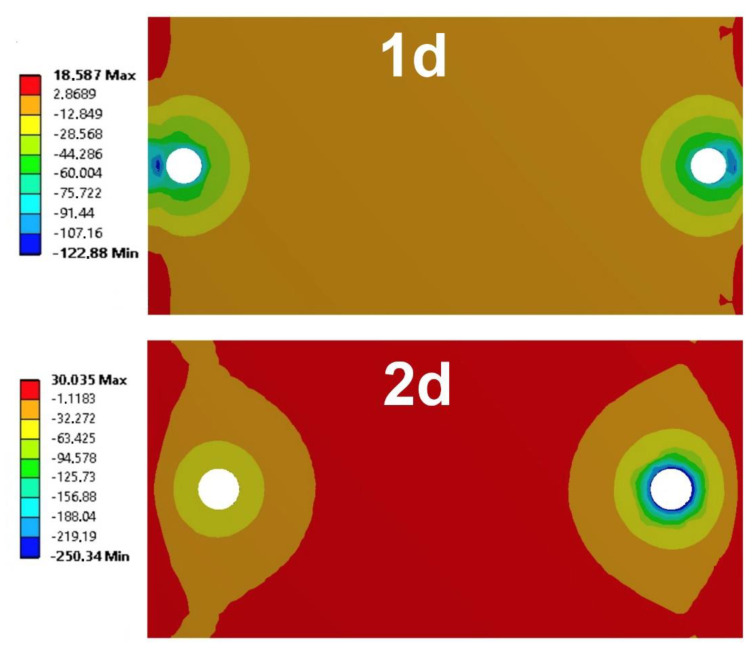
Maps of normal stress distribution for arrangement of mechanical fasteners in one hole diameter (1d) and two hole diameters (2d).

As a result of the calculations, i.a. stress maps in the adhesive layer were defined. The maximum values of shear stress occurred at the holes, while at the edges of the overlap, the highest values of maximum principal stress and normal stress perpendicular to the adhesive layer were noted—these are a measure of the peel phenomenon occurring in this type of joint. Maps of the normal stress distribution in the adhesive layers that causes the peeling phenomenon for two assembly methods of mechanical fasteners (1d and 2d arrangement) are presented in Figure 6.

**Figure 7:**
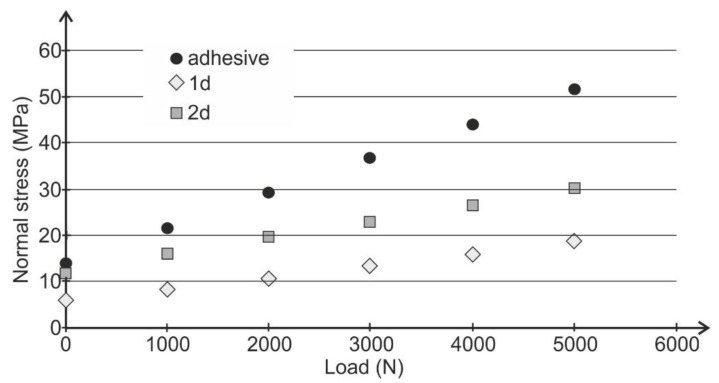
The maximum normal stress causes peeling dependence on the load for the model of adhesive and hybrid joints in the 1d and 2d arrangements.

As the fasteners were moved closer to the edge of the overlap, the maximum normal stress values decreased. Using the prepared model, calculations were made for smaller load values and the adhesive joint as well. The values of the maximum normal stresses perpendicular to the adhesive layer as a function of the load for the adhesive joint and hybrid joints in the 1d and 2d arrangements are presented in Figure 7. The results confirm that when the mechanical fasteners are displaced towards the edge of the overlap, the peeling phenomenon for all implemented load values reduces. In addition, an increase in the load capacity and fatigue life of hybrid joints can be observed.

The numerical calculations show that the use of mechanical fasteners in hybrid lap joints reduces the value of normal stresses in the adhesive layer, which results in adhesive failure of the joint at higher loads (increased joint strength). Therefore, locating mechanical fasteners closer to the edge of the lap reduces normal stresses to a greater extent.

### 3.2. Results and Discussion of Experimental Research

**Figure 8:**
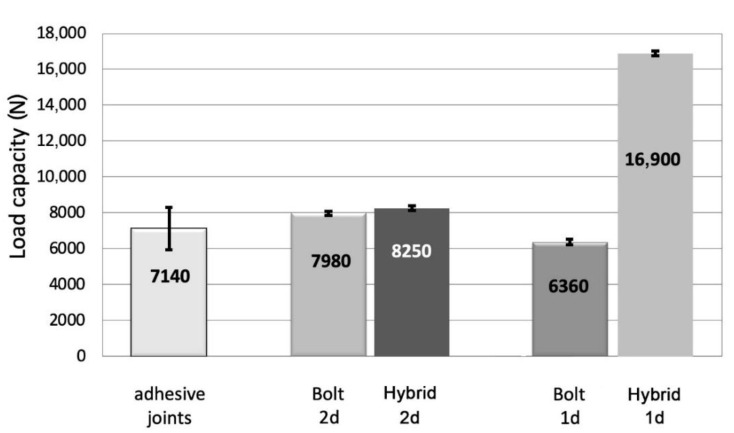
Comparison of the load capacity of the adhesive, mechanical, and hybrid joints for aluminum alloy samples and two mechanical fastener assembly methods (i.e., 1d and 2d).

The results of the static and fatigue tests for the first stage, carried out with aluminum alloy specimens, are presented in Figure 8 as well as in Table 4.

**Figure 9:**
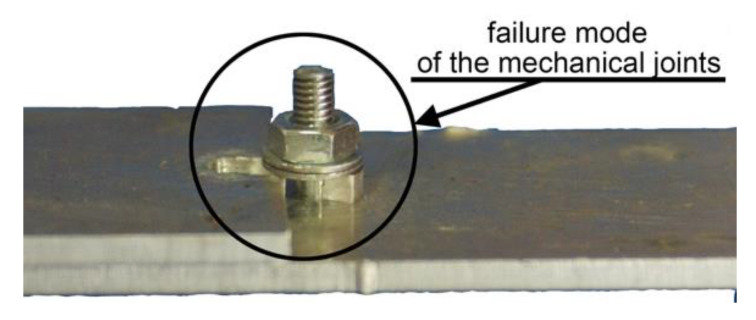
Failure of the mechanical joint as a result of exceeding the surface pressure (static test).

In the case of joining metal parts together, the load capacity of both the adhesive and the mechanical joints was comparable. The load capacity of the mechanical and hybrid joints significantly depended on the configuration of the fasteners’ assembly. If the mechanical fasteners were mounted at a distance of 1d, an approximately 20% decrease in the load capacity of the joint was noticed in comparison with a 2d distance (Figure 8). It should be emphasized that for a 1d assembly distance, there was a different mechanism of joint damage—the bolts were not sheared at a 2d distance, but the failure was a result of exceeding permissible stresses on the surface pressures in the bolt holes (Figure 9).

When comparing the load capacity of the hybrid and mechanical joints, it was found that only in variant 1d was it possible to obtain over a 2.5-fold increase in the load capacity of the hybrid joint compared to bolt joint (Figure 8). For the assembling fasteners at a 2d distance, the increase in the load capacity of the hybrid joint was hardly noticeable—approximately 3% (Figure 8).

**Figure 10:**
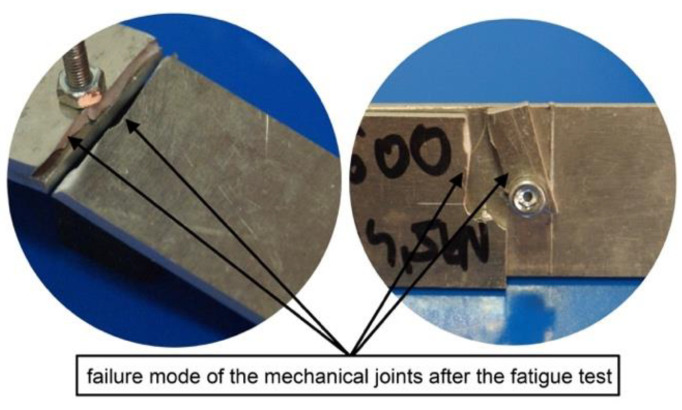
Photographs of the damaged mechanical joints after the fatigue tests.

The modification by which fasteners were placed closer to the edge of the lap was successful in terms of the increased fatigue life of both the mechanical and hybrid joints (Table 4). In the case of mechanical joints, the change in the assembly method of fasteners from variant 2d to variant 1d resulted in more than a two-fold increase in fatigue life. This increase in durability could have resulted firstly from the lower value of secondary bending moments that occurred in the critical section, and secondly from another form of fatigue damage to the joints. The mechanical joints were damaged due to fatigue fracture as a result of crack propagation, not along the critical section, but at the lap edges (Figure 10).

The modification of the fasteners’ placement in the case of the hybrid joints also influenced the fatigue life. The joints with 1d variant had an average fatigue life over four times higher than the hybrid joints in variant 2d and an approximately 10 times higher fatigue life compared to the mechanical joints in variant 1d (Table 4). The hybrid specimens in variant 1d, marked as numbers 1 and 4, were not damaged during the tests (after exceeding 5 million cycles, the durability tests were stopped).

**Figure 11:**
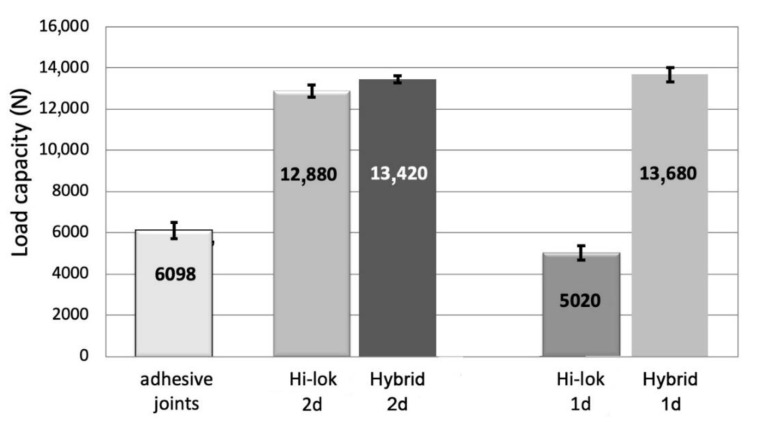
Comparison of the load capacity of the adhesive, mechanical, and hybrid joints for the composite samples and two mechanical fasteners assembly variants (i.e., 1d and 2d).

The results of static and fatigue tests of the CFRP specimens are presented in Figure 11 and Table 5.

The average value of the load capacity of the adhesive joints of the CFRP specimens was approximately 15% lower than the load capacity of the aluminum alloy specimens (Figure 8 and Figure 11). The lowest load capacity in the joints of the CFRP elements is characteristic of Hi-lok fasteners in variant 1d (Figure 11). Additionally, adhesive joints demonstrate a low load capacity compared to the other examined joints: It was higher by only 20% than in the case of the Hi-lok 1d joints. The load capacity of the remaining tested joints was more than twice that of the load capacity of the adhesive joints, whereas the highest average load capacity was observed for the 1d hybrid joints (Figure 11).

Likewise, as in the case of the joints of the aluminum alloy elements, the load capacity of the mechanical joints significantly depended on the configuration of the fasteners’ assembly. The assembly of fasteners close to the edge lap (1d) resulted in joints with a load capacity that merely equaled 39% of the capacity of the mechanical joints in variant 2d (Figure 11).

**Figure 12:**
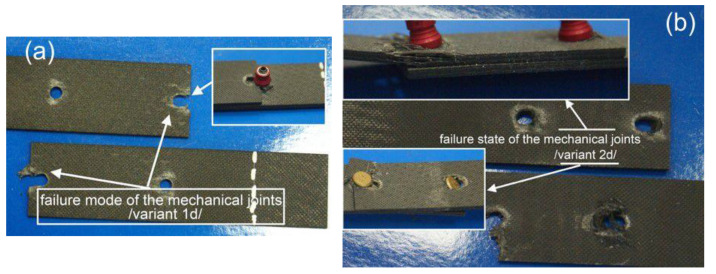
Photographs of the damaged mechanical joint in variants 1d (**a**) and 2d (**b**).

In contrast to the aluminum samples, irrespective of the fasteners’ assembly, the composite material of the joined elements underwent degradation and damage, which is presented in Figure 12.

In the hybrid joints, it was possible to observe an increase in the load capacity in relation to the mechanical joints, particularly in the case of the joints assembled in variant 1d—by more than 2.5 times (Figure 11). The hybrid joints in variant 2d had a load capacity that was only approximately 10% higher in relation to the mechanical joints. In the hybrid joints in variants 1d and 2d, the differences in load capacity were 2% (Figure 11).

In relation to the fatigue tests of the CFRP specimens, there were no clear traces of fatigue damage in the mechanical and hybrid joints, as was the case in the aluminum alloy specimens (Table 5). Only adhesive joints were damaged by the separation of the bonded specimens. The research into other joints was interrupted before any clear fractures were observed. In order to assess the effectiveness of the proposed modifications (displacement of the mechanical fasteners in the hybrid joints), the tomographic method was used.

**Figure 13:**
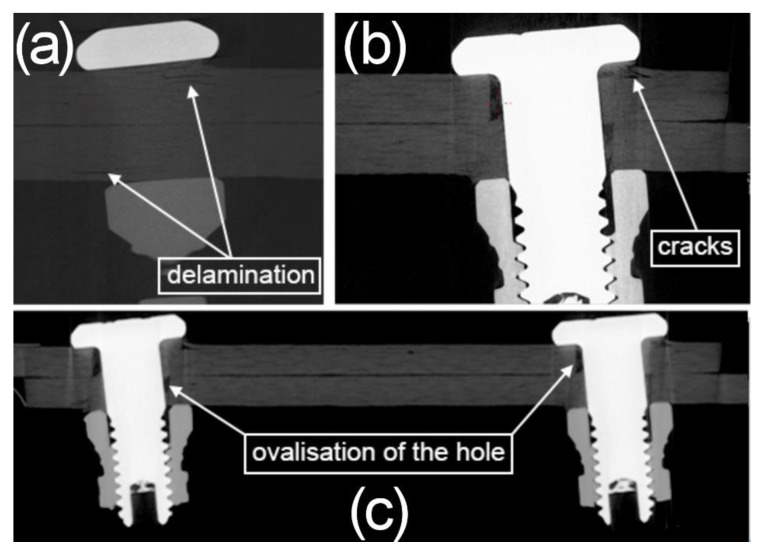
Tomographic images of the process of damage to the composite material in a mechanical joint using the Hi-lok 2d assembly variant (specimen after durability tests of 618,700 cycles, the range of load in cycle 6 ÷ 10 kN). (**a**) delaminations, (**b**) cracks, (**c**) ovalisations of the assembly holes.

**Figure 14:**
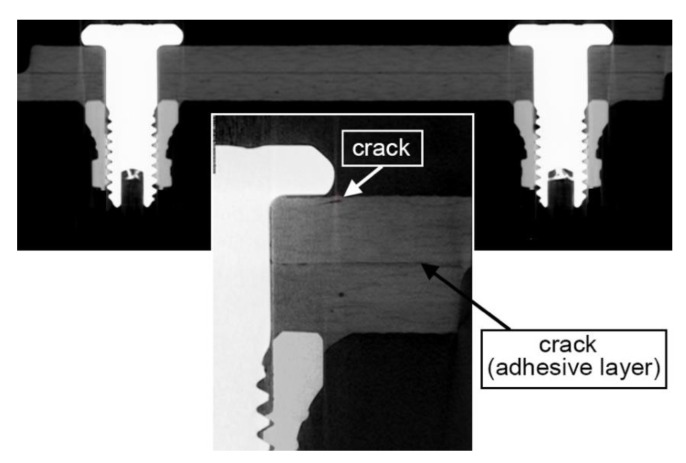
Tomographic images of a crack in the composite material and in the adhesive layer of a hybrid joint. The Hi-lok 2d assembly variant was used (specimen after durability tests of 2,825,500 cycles, range of loads in cycle 6 ÷ 10 kN).

In the tomographic images of the examined joints, three types of damage can be found (Figure 13 and Figure 14):

- Delamination of the connected material;
- Cracks;
- Ovalisation of the assembly holes.

The authors did not notice any damage to the mechanical fasteners in any of the tomograms.

In the mechanical joints (Figure 13), due to fatigue loads, there was deformation of the assembly holes (ovalisation) (Figure 13c), as a result of which the joints deviated axially and the joined elements shifted against one another. Figure 13a presents a cross-section of the joined material in front of the fastener, in which there is clear delamination of the upper composite layers. The delamination was possibly caused by the synergistic action of the local bending of the specimen under the pressure of the axially deviated joint and the pressure of the opening onto the side surface of the fastener and the fastener nut on the surfaces of the jointed elements, as well as peeling, which resulted from the stress concentrations at the edge of the lap. It seems that because of these factors, the initial delamination may have developed into a crack due to bending of the separated layer (Figure 13b).

The tomographic images of the hybrid joint in variant 2d show small cracks located where the edge of the fastener head presses onto the assembled component. The crack, noticeable in Figure 14, is shallow and extends deep into the material to the thickness of a single layer. Moreover, in the vicinity of the lap edge, it is possible to notice the cracking of the adhesive joint, which is the result of stress concentration at the ends of the lap joint.

**Figure 15:**
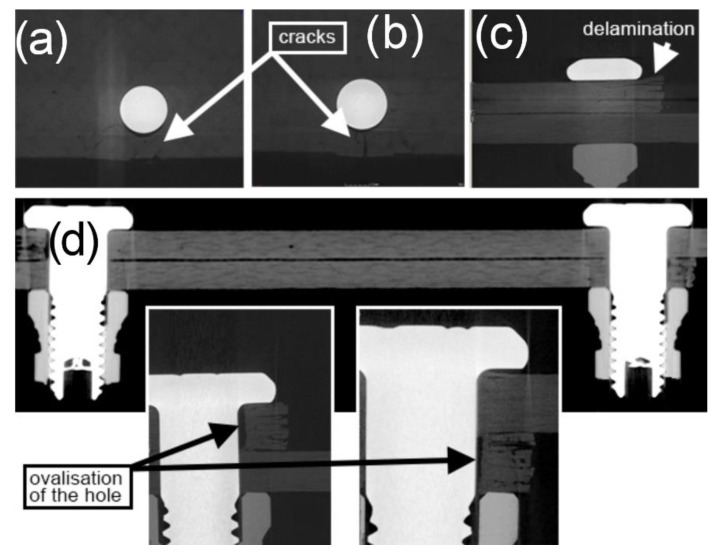
Tomographic images of cracks on the composite material in a mechanical joint. The Hi-lok 1d assembly variant was used (specimen after durability tests of 882,500 cycles, range of loads in cycle 6 ÷ 10 kN). (**a**) and (**b**) cracks, (**c**) delamination, (**d**) ovalisations of the assembly holes.

In the mechanical joints of variant 1d, it is possible to observe similar phenomena to those in the 2d mechanical joints. Due to fatigue loads, there was ovalisation of the assembly holes (Figure 15d) as a result of the deformation of the material. It must be stressed that the value of ovalisation is smaller than that in the 2d joints, which is associated with a decreased value in the load in the cycle. As a result of the ovalisation of the holes, the joints swung axially, which caused a local accumulation of surface stress. On both edges of the laps, there is clear, deep delamination over the entire width of the lap, extending into the wall of the assembly hole, as well as cracks in the individual layers toward the direction of the lap edge (Figure 15a,b).

**Figure 16:**
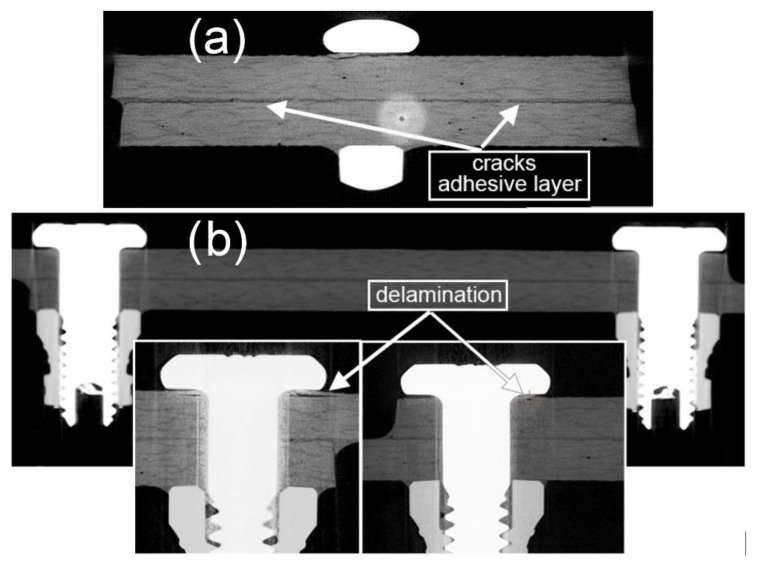
Tomographic images of cracks in the composite material and the adhesive layer of a hybrid joint, using the Hi-lok 1 d assembly variant (specimen after durability tests of 2,902,100 cycles in the range of loads in cycle 6 ÷ 10 kN). (**a**) Cracks in adhesive layer, (**b**) delaminations.

In the tomographic images of the cross-section of the hybrid joint, cracks can be observed along approximately two-thirds of the width of the lap (Figure 16a). However, they occurred in a cross-section approximately 2 mm away from the assembly hole, which indicates substantial degradation of the adhesive joint. In the longitudinal section, such cracks in the vicinity of the mechanical fasteners cannot be found (Figure 16b). Close to the heads of the mechanical fasteners, it is possible to observe delamination of the joined material (Figure 16b). However, the nature of both the cracks and delamination may indicate that their root cause could have arisen during preparation of the joint.

The results of the studies presented are in line with the results of the study in publications that were analyzed by the authors in the introduction to this study. However, it should be noted that the added value of the presented research was a comprehensive presentation of the impact of the original modification of the location of the connector (variant 1d vs variant 2d) on the load capacity and fatigue life of mechanical and hybrid connections for two different types of materials, i.e., aluminum and composite. The presentation of quantitative differences in the load capacity and fatigue life of adhesive, mechanical and hybrid joints for the modification proposed by the authors, consisting in shifting the connectors towards the edge of the joint, is something new among the analyzed publications, which deal with the issues discussed here in a narrower scope, e.g., testing the joints of one type of material or the arrangement of connectors other than the one analyzed here.

## 4. Conclusions

Based on the above research, the following conclusions can be drawn:

1. Hybrid adhesive–mechanical joints should be designed in such a way that the failure of the adhesive layer and the mechanical connectors occurs simultaneously. However, the failure of the adhesive joint typically occurs earlier, which results in a lack of increase in the strength of the hybrid joint compared to the mechanical joint;
2. The failure of an adhesive joint may be of an adhesive, cohesive, or mixed cohesive–adhesive nature. Normal stresses perpendicular to the surface of an adhesive joint are responsible for the adhesive failure. Lowering the value of these stresses increases the strength of the adhesive joint. The authors hypothesized that locating the fasteners closer to the edge of the joint should result in a reduction in normal stresses in the adhesive layer due to the pressure of the heads of the mechanical fasteners. This hypothesis was confirmed by the FEM calculations based on a simple model, which took into account the pressures of the connectors’ heads;
3. Experimental studies have shown that placing mechanical connectors at a distance of 2d (d = connector diameter) from the edge in hybrid joints does not increase the strength of such joints in relation to mechanical joints. Placing mechanical connectors at a distance of 1d from the edge reduces the strength of mechanical joints (to a greater extent when composite elements are joined) but increases the strength of hybrid joints;
4. The tested hybrid joints had a significantly greater fatigue life compared to the mechanical and adhesive joints, regardless of the arrangement of said mechanical joints. In the case of joining sheets of aluminum alloy, locating the fasteners closer to the edge (option 1d) resulted in an almost four-fold increase in fatigue life in relation to option 2d;
5. Modifying the spacing of mechanical connectors was not found to have an effect on the fatigue life of hybrid joints in the case of joining composite elements. This was probably due to the use of Hi-lok fasteners, which, due to the large diameters of their shanks’ heads, exerted a greater pressure on the joined elements.

## Figures and Tables

**Table 1:** Values of the parameters of the materials used in the calculations (alloy 2024 and Epidian 57Z1 data taken from the authors’ own experiments, and that of steel from the library).

Material	Parameter	Value
Aluminum alloy 2024 (adherends)	E (MPa)	72,000
	ν	0.3
	R_02_ (MPa)	330
	Tangent Modulus (MPa)	1200
Epidian 57/Z1 adhesive	E (MPa)	2000
	ν	0.35
Steel (fasteners)	E (MPa)	200,000
	ν	0.3

**Table 2:** Contact elements defined for tested specimen.

Adjacent Items	Adherends–Adhesive	Head of Fasteners–Adherends	Fasteners Shanks–Adherends and Adhesive	Sleeves-Adherends	Head of Fasteners-Shanks
Contact type	Bonded	Frictional, *f* = 0.2	Frictional, *f* = 0.2	Frictional, *f* = 0.2	Bonded

*f*—friction coefficient.

**Table 3:** Parameters of the fatigue tests.

Load Scope in a Cycle			
Type of Material	Aluminum Alloy	Composite	
**Type of joint**	adhesive (1d and 2d), mechanical (1d and 2d), and hybrid (1d and 2d)	mechanical (2d) and hybrid (1d and 2d)	mechanical (1d)
**Load cycle—min ÷ max (average) (kN)**	1.5 ÷ 4.5 (3)	6 ÷ 10 (8)	1 ÷ 4 (2.5)
**Frequency of load (Hz)**	8	8	8
**Coefficient of cycle asymmetry**	0.33	0.6	0.25

**Table 4:** Fatigue life of aluminum alloy joints.

Fatigue Life (Number of Cycles)					
Series of Joint	Adhesive	Mechanical (2d)	Hybrid (2d)	Mechanical (1d)	Hybrid (1d)
1	7000	141,550	1,059,750	472,600	5,010,650 *
2	9500	89,300	1,260,500	281,600	2,247,800
3	4300	166,800	1,015,400	450,500	3,945,100
4	12,000	179,200	1,098,500	n/a	5,087,500 *
Average	8200	162,517	1,108,538	401,567	4,072,763

* Specimen was not damaged.

**Table 5:** Fatigue life of joints with the carbon fiber-reinforced polymer (CFRP) elements.

Fatigue Life (Number of Cycles)					
Type of Connection	Adhesive	Mechanical (2d)	Hybrid (2d)	Mechanical (1d)	Hybrid (1d)
1	400	500,000 ^1^	2,500,000 ^1^	700,000 ^1^	2,500,000 ^1^
2	800	600,000 ^1^	2,750,000 ^1^	800,000 ^1^	2,750,000 ^1^
3	2550	618,700 ^1,^*	2,825,500 ^1,^*	882,500 ^1,^*	2,902,100 ^1,^*

^1^ No visible fatigue failure of the specimen. * The specimens were tomographically tested.
